# Establishment of an optimized chemotherapy-induced mouse model for premature ovarian failure: protocol and findings

**DOI:** 10.18632/aging.206332

**Published:** 2025-10-28

**Authors:** Negar Yavari, Narges Zaeemzadeh, Behrouz Gharesi-Fard, Negar Ajabi Ardehjani, Tayebeh Rastegar, Fardin Amidi

**Affiliations:** 1 Department of Anatomy, School of Medicine, Tehran University of Medical Sciences, Tehran, Iran; 2 Department of Reproductive Biology, School of Advanced Medical Sciences and Technologies, Shiraz University of Medical Sciences, Shiraz, Iran; 3 Department of Immunology, Shiraz University of Medical Sciences, Shiraz, Iran; 4 Department of Anatomical Sciences, School of Medicine, Guilan University of Medical Sciences, Rasht, Iran; 5 Department of Infertility, Yas Hospital, Tehran University of Medical Sciences, Tehran, Iran

**Keywords:** Busulfan, Cyclophosphamide, mice, premature ovarian failure

## Abstract

Objective: The aim of this study was to induce a practical premature ovarian failure (POF) mouse model using Cyclophosphamide (CTX) and Busulfan (Bu), considering both drug exposure duration and natural recovery time at the optimal dose.

Methods: Female NMRI mice (6-8 weeks) received single intraperitoneal injections of four CTX/Bu dose regimens. Controls were injected with a single dose of equal volume of saline (n=3/group). To evaluate natural ovarian recovery, treated mice were left without intervention for 3 and 4 weeks after the chemotherapeutic combination administration. In addition, follicle counting (in all groups) and hormonal analyses (in the optimal group) were performed to validate the recovery and model.

Results: Among all doses, the CTX 100 mg/kg + Bu 20 mg/kg regimen reliably induced POF within 3 weeks post-administration, as demonstrated by three key criteria: (1) persistent follicular decline in ovarian reserve (2) endocrine disruption (significantly elevated FSH and suppressed AMH/E2 levels and (3) sustained ovarian dysfunction throughout the 3-week post-induction observation period (until week 6 post-injection). No spontaneous ovarian recovery was observed during the 3-week post-induction period. Notably, the treatment protocol showed excellent safety profiles so that no mortality was observed compared with controls.

Conclusions: These results suggest that the single dose IP injection of the CTX 100 mg/kg + Bu 20 mg/kg can effectively induce POF within 3 weeks post-administration and POF model maintains for at least 3 weeks after induction.

## INTRODUCTION

Premature ovarian failure (POF), also known as premature ovarian insufficiency (POI), represents a significant challenge in the field of reproductive medicine that affects approximately 0.01% of women at age 20, 0.1% at age 30, and 1% at age 40. Still, its prevalence is steadily increasing [1–3]. POF is usually accompanied by amenorrhea, hypergonadotropism, and hypoestrogenism, a process that is accompanied by decreased estradiol production and increased FSH up to the menopause threshold before the age of 40 [2, 4]. Also, POF contributes to a range of long-term health consequences, including night sweats, insomnia, mood changes, blood pressure fluctuations, inattention, osteoporosis, cardiovascular disease, and psychological distress [5, 6]. POF has a significant impact on the overall health and quality of life of affected women. The etiology of POF remains unknown and is related to multifactorial, encompassing genetic, infection, autoimmune, idiopathic, and iatrogenic causes [7].

Chemotherapy-induced ovarian failure is a prevalent issue faced by cancer survivors and one of the critical causes of iatrogenic POF [8]. Today, animal models are essential instruments for investigating pathogenesis and precise mechanisms of diseases. Therefore, various efforts have been made to build an appropriate model for POF to permit rigorous experimental research into the underlying mechanisms and examine potential therapeutic strategies [9]. Animal models for POF employ four main approaches to reflect the pathologies causing the condition. These include chemotherapy-induced models [10], surgical models like ovariectomy [11], autoimmune models [12, 13], and gene knockout models [14]. Each method has its advantages and limitations for studying POF.

In this study, we present a protocol for inducing POF in NMRI mice via intraperitoneal injection of two chemotherapeutic agents: Cyclophosphamide(CTX) and Busulfan(Bu). Bu and CTX are widely used chemotherapeutic agents that cause POF in mice by causing follicular destruction and oxidative damage [15]. Various animal models using alkylating agents such as CTX and Bu have been developed to study the pathophysiology of chemotherapy-induced ovarian damage [16].

These traditional chemotherapy medicines, which are frequently used to cure cancer, have substantial reproductive damage and are utilized to induce POF models in animals [17, 18]. CTX is a gonadotoxic agent, causing ovarian dysfunction through damage to the ovary and decreased ovarian reserve [19]. It mediates its effects through forming DNA crosslinks, leading to the apoptosis of rapidly dividing cells, including ovarian follicles [20].

Bu is a cytotoxic agent that acts on ovarian germ cells and exacerbates follicular depletion [21]. Considering that the mechanism of action of CTX is through the induction of apoptosis and the PI3K-AKT pathway, and Bu leads to the depletion of primordial reserves by inducing apoptosis in oocytes and granulosa cells. In addition, CTX metabolites lead to excessive activation of primordial follicles, which ultimately lead to atresia and a decrease in ovarian follicular reserve [15, 22–26].

The combination of these two agents produces a synergistic action and effectively models the clinical situation of chemotherapy-induced POF. This combined treatment affects the ovaries extensively. While according to studies, it has less effect on the spleen, lungs and kidneys and has and exerts minimal to no effects on the heart, stomach, or pancreas. Therefore, this combination can be a good choice because it causes fewer side effects, making it a suitable platform for studying specific ovarian damage without systemic complications [10, 23, 27]. However, despite the established utility of this model, a critical limitation persists: substantial variability in reported dosing regimens with no consensus on an optimal protocol that balances efficacy with minimal systemic toxicity [19, 20, 21]. Existing studies have primarily focused on maximizing follicular depletion. Unlike patients in clinical settings, where complete follicular depletion is uncommon and typically a gradual decline in ovarian reserve occurs, establishing an appropriate dosing regimen for POF induction in animal models becomes significantly more challenging [28]. A critical gap remains in identifying the lowest effective CTX/Bu dose that reliably induces POF while: minimizing extra-gonadal toxicity (e.g., body weight loss), maintaining ovarian stromal viability for subsequent investigations into follicular atresia mechanisms, and standardizing protocols to enhance reproducibility across studies. Furthermore, the optimal model in the present study provides a platform for exploring the broader implications of ovarian dysfunction. This research aimed to examine the effects of different CTX and Bu doses on ovary function over time. The findings from this study hold significant promise for improving the reproductive and overall health outcomes of cancer survivors, thereby enhancing their quality of life and long-term well-being.

## MATERIALS AND METHODS

### Experimental animals

All 30 experimental NMRI female mice (6 to 8 weeks old, 24-26g) were purchased from Royan Institute and housed in a temperature- and humidity-controlled animal facility under a 12-hour light-dark cycle with food and water available ad libitum. The mice were allowed to acclimatize for 1 week. All experiments were performed in accordance with the Institutional Animal Care Committee of Tehran University of Medical Sciences (IR.TUMS.AEC.1402.047).

### Establishment of POF model

To induce POF, 24 NMRI female mice were randomly divided into four groups (n: 6 mice per group) and treated with different combined doses of CTX and Bu via intraperitoneal injection and six mice were assigned to the vehicle-treated control group [27].

- Group 1: 80 mg/kg body weight (bw) CTX and 15 mg/kg bw Bu
- Group 2: 100 mg/kg bw CTX and 20 mg/kg bw Bu
- Group 3: 120 mg/kg bw CTX and 12 mg/kg bw Bu
- Group 4: 120 mg/kg bw CTX and 30 mg/kg bw Bu

The control group was injected with an equal volume of saline for 3 and 4 weeks. CTX and Bu were diluted in normal saline. Mice were monitored for three weeks post-injection. Mortality was checked daily, and body weight was measured. Additionally, signs of adverse effects (e.g., lethargy, reduced mobility, or local reactions) were tracked throughout the observation period.

### Tissue harvesting and sample collection

To assess the induction of POF model, following three- and four-week observational periods after a single intraperitoneal administration of CTX and Bu all experimental and control mice were anesthetized via intraperitoneal injection of 1% sodium pentobarbital (50 mg/kg) and euthanized by cervical dislocation. Bilateral ovaries were immediately excised and immersion-fixed in 10% neutral-buffered paraformaldehyde (PFA; pH 7.4) for 24-48 hours to ensure optimal tissue preservation. Fixed specimens were then processed through graded ethanol series, embedded in paraffin, and sectioned coronally at 5 μm thickness using a rotary microtome for subsequent histological analyses.

### Hematoxylin–Eosin staining and follicle counting

At the end of the modeling period (week 3 and 4 post injection), the ovaries were extracted, washed roughly with PBS, and placed in 10% paraformaldehyde overnight at room temperature for 48 hours before being embedded in paraffin and serially sectioned at a thickness of 5 μm for further analysis. Following sectioning, fixation, deparaffinization, dehydration, and clearing, Hematoxylin-Eosin (HE) staining was performed, according to the manufacturer’s instructions [29]. Ovarian follicle quantification was conducted on serial 5-μm hematoxylin and eosin (H&E)-stained tissue sections using light microscopy at 4x and 10x magnification (Olympus CX31). To maintain objectivity, two independent, blinded investigators performed all follicular assessments. The counting protocol incorporated several quality control measures:

1. Systematic random sampling was implemented by analyzing every fifth section (50 μm intervals) to prevent follicle duplication across sequential sections;
2. Enumeration commenced at a standardized 12 o'clock position with clockwise progression through the microscopic field;
3. Strict inclusion criteria were applied, requiring clear visualization of the oocyte nucleus for follicle registration;
4. Developmental staging followed established morphological parameters, classifying follicles as primordial, primary, secondary, or antral based on granulosa cell architecture and antral space formation.

The ovarian reserve, which is assessed by primordial follicles in the ovarian cortex, and the stages of follicles were determined based on their morphology and granulosa cell structure. Following H&E staining, the follicles were categorized into five groups based on their development stages [30]:

Primordial Follicle (Type 1): One layer of flattened granulosa cells surrounding the oocyte.

Primary Follicle (Type 2): One to two complete layers of cuboidal granulosa cells surrounding the oocyte.

Secondary Follicle (Type 3): An oocyte surrounded by more than one layer of cuboidal granulosa cells with no visible antrum.

Antral Follicle (Type 4): An oocyte surrounded by multiple layers of cuboidal granulosa cells containing one or more antral spaces, possibly with a cumulus oophorus and thecal layer.

Atretic Follicle (Type 5): A follicle that enters a degenerative process without ovulation.

### Serological analysis

Following histomorphometric determination of the optimal CTX/Bu regimen for POF induction, endocrine validation of the model was performed through quantitative serum analysis of follicle-stimulating hormone (FSH), estradiol (E2), and anti-Müllerian hormone (AMH) using standardized ELISA protocols six weeks post injection (in order to confirmation of POF model maintenance). Terminal blood samples were collected via transcardiac puncture (27G needles) under deep anesthesia, clotted for 20 min at room temperature, and centrifuged (3000 × g, 10 min). Resultant serum aliquots were cryopreserved at -80° C until batch analysis. Endocrine profiling was performed on the CTX 100 mg/kg + Bu 20 mg/kg cohort and compared with vehicle-treated controls to biochemically validate successful POF induction. Commercial ELISA kits were employed with the following specifications:

FSH: (Cat No:EM1035, Finetest, USA)

E2: (Cat No:KGE014, R&D, USA)

AMH: (Cat No:MBS2507173, MyBioSource, USA)

### Confirmation of POF model induction

The successful induction of POF model was confirmed through a comprehensive evaluation of histopathological alterations, morphometric changes, and follicular quantification.

### Confirmation of POF model maintenance and natural recovery assessment

To determine the time it takes for ovarian natural recovery to ensure that ovarian dysfunction and POF model remain stable for at least three weeks (The 3-week endpoint aligns with the typical duration of folliculogenesis in mice (~21 days) [31], the treated mice with optimal dose of CTX/Bu were under observation without further intervention for periods of 3 and 4 weeks following the induction of the POF model (6 and 7 weeks post single intraperitoneal injection of CTX/Bu). Subsequently, the restoration of their ovarian functions was evaluated through a detailed histological examination of the ovarian tissues and follicle counting, and also biochemical assessment was performed for serum FSH, E2, and AMH levels.

### Statistical analysis

All statistical analyses were performed using GraphPad Prism software (version 10; GraphPad Software, San Diego, CA, USA). Data are presented as mean ± standard deviation (SD). For body weight and follicle count analyses, two-way analysis of variance (ANOVA) was applied with factors of treatment group and time, followed by Tukey’s post-hoc multiple comparisons test. For hormonal assays (AMH, E2, and FSH), comparisons between control and treated groups were conducted using unpaired two-tailed Student’s t-tests. Statistical significance was set at p < 0.05.

### Data availability

This published article contains all of the data collected or analyzed during this investigation.

## RESULTS

**Figure 1:**
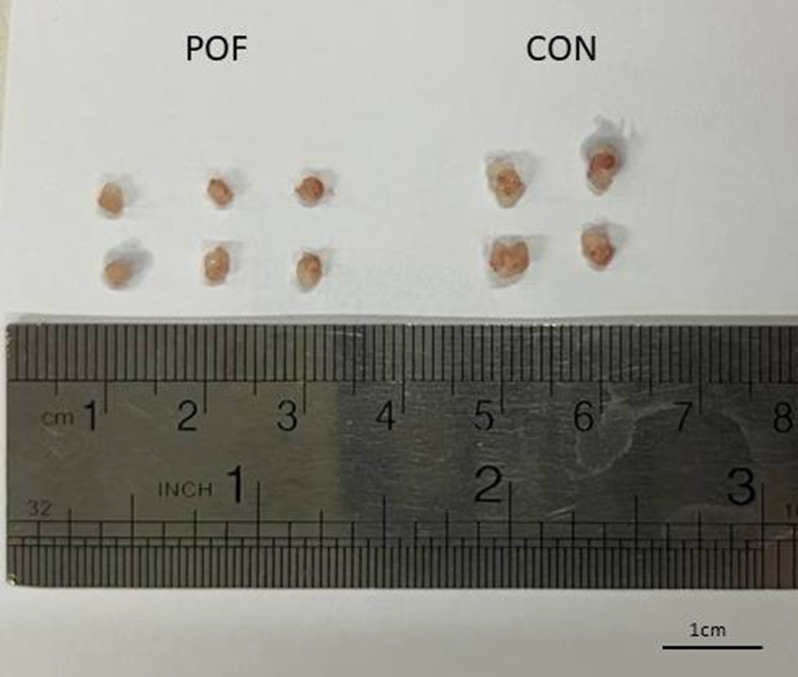
The ovary of CTX/BU-treated mice (left) was smaller than that of the controls (right).

**Figure 2:**
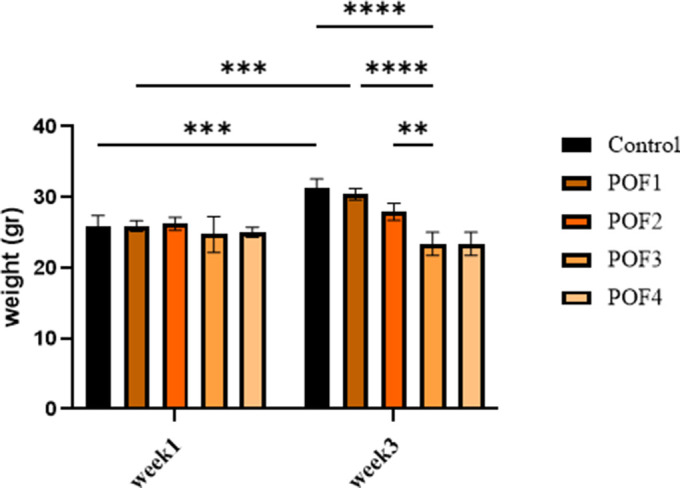
**Body weight of mice at week 1 (first day post-administration) and week 3 (the twenty-first day post-administration) across different treatment groups: Cyclophosphamide (CTX:80,100,120,120 mg/Kg).** Data are presented as mean ± SD. Two-way ANOVA revealed significant effects of time, group, and time × group interaction. Post-hoc analysis indicated significantly lower body weights in POF2, POF3, and POF4 groups compared with controls at week 3 (*p < 0.05).*

All mice survived the entire observation period, with no mortality recorded during the initial three-week post-administration phase or the extended follow-up (weeks 9–12). Comparative analysis of ovarian morphology, as presented in Figure 1, demonstrated a significant reduction in ovarian size within the optimal dose of (CTX 100 mg/kg + Bu 20 mg/kg) cohort compared to the control specimens. Body weight was also measured on the first and the twenty-first days post-administration (due to the importance of folliculogenesis in mice). These results indicate that body weight was influenced both by the experimental group and by time, and that weight changes over time differed among groups. Post-hoc multiple comparisons further demonstrated that body weight gain observed in the control group between week 1 and week 3 was significantly blunted in chemotherapy-treated groups. In particular, the POF2 group showed a significantly lower body weight compared with the control group at week 3 (mean difference ≈ 4–5 g; p < 0.05). Similar trends were observed in the higher-dose groups (POF3 and POF4), which also exhibited reduced weight compared with controls, whereas no significant difference was detected in the lowest-dose group (CTX 80 mg/kg + Bu 15 mg/kg) These findings indicate that CTX/Bu treatment impairs the normal weight gain trajectory in a dose-dependent manner (Figure 2).

**Figure 3:**
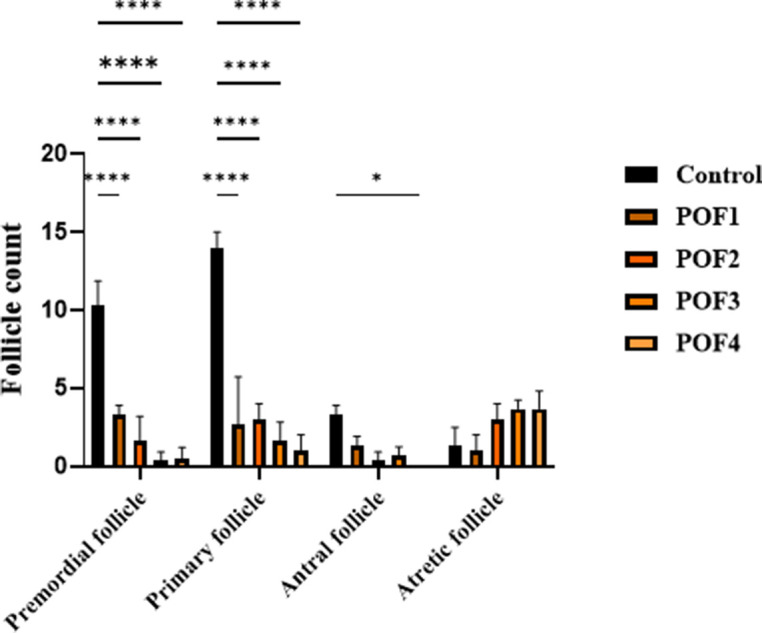
**Three weeks post-induction, follicle counts were analyzed in CTX/Bu-treated mice, using Cyclophosphamide (CTX: 80, 100, 120, 120 mg/kg) in combination with Busulfan (Bu: 15, 20, 12, 30 mg/kg).** This design enabled dose-dependent assessment of ovarian follicular depletion in the POF mode.

Clinical monitoring detected no signs of distress (e.g., lethargy, ruffled fur) or adverse effects (e.g., local inflammation, behavioral changes) at any timepoint. These findings collectively demonstrate the safety and tolerability of the treatment regimen in this model. The follicular count within the ovarian cortex serves as a critical marker of ovarian reserve. The statistical outcomes of follicle quantification in mice, assessed three weeks after model induction, are summarized below. Two-way ANOVA revealed that follicle composition was significantly influenced by both group and follicle type, with a significant interaction between these factors (*p* < 0.0001 for all). Compared with controls, the numbers of primordial and primary follicles were markedly reduced in all chemotherapy-treated groups (all Tukey-adjusted *p* < 0.0001). Antral follicles were significantly decreased in POF2 and POF4 (*p* ≈ 0.02–0.03), while POF3 showed a borderline reduction. Although atretic follicles tended to increase and were exacerbated at higher doses (POF3 and 4), these changes did not reach statistical significance after adjusting for multiplicity (Figure 3).

**Figure 4:**
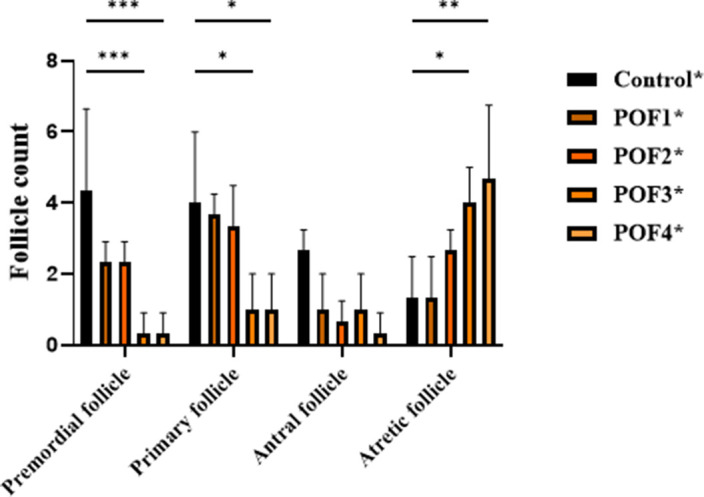
**4 weeks post-induction, follicle counts were analyzed in CTX/Bu-treated mice, using Cyclophosphamide (CTX: 80, 100, 120, 120 mg/kg) in combination with Busulfan (Bu: 15, 20, 12, 30 mg/kg).** This design enabled dose-dependent assessment of ovarian follicular depletion in the POF model.

In the second group, mice were assessed 4 weeks after model induction. Two-way ANOVA demonstrated significant main effects of group, follicle type, and their interaction (all *p* < 0.05). Tukey’s multiple comparisons revealed that primordial follicles were markedly reduced in POF3 and POF4 groups compared with controls (*p* = 0.0009), while POF1 and POF2 showed non-significant decreases. Similarly, primary follicles were significantly decreased in POF3 and POF4 compared with controls (*p* = 0.0191), with additional differences observed between POF2 and POF1 as well as between POF4 and POF2 (*p* ≈ 0.0469). In contrast, antral follicles did not differ significantly among groups. Notably, atretic follicles were significantly increased in POF3 (*p* = 0.0469) and POF4 (*p* = 0.0072) relative to controls. These findings indicate that higher CTX/Bu doses exacerbate follicular depletion and atresia after 4 weeks, with POF3 and POF4 showing the most severe phenotypes (Figure 4).

**Figure 5:**
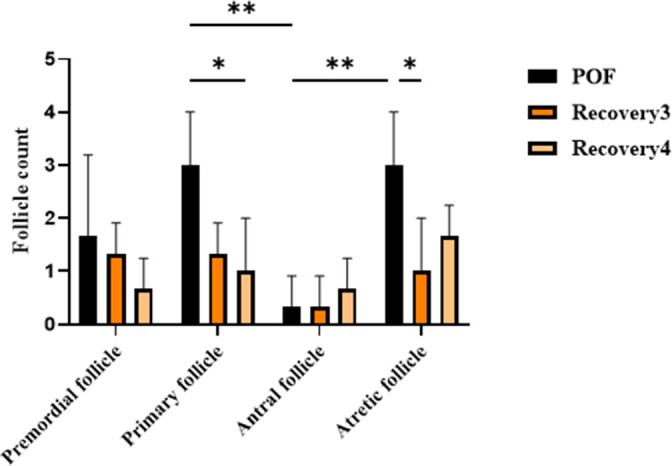
**Follicle counts in the optimal dose group (CTX 100 mg/kg + Bu 20 mg/kg) at day 21 and during 3- and 4 weeks post-induction (natural recovery).** Two-way ANOVA showed significant effects of follicle type and group (*p* < 0.05), but no significant interaction. Both recovery groups exhibited persistently reduced primordial, primary, and antral follicles, with sustained atresia, confirming the stability of the POF model up to 4 weeks post-induction.

This severe phenotype, characterized by profound follicular depletion, diverges from the clinical presentation observed in patients with POF. In affected women, POF is typically associated with a marked reduction of the ovarian reserve; however, a limited number of primordial or primary follicles generally remain [28]. This observation underscores the role of combined CTX and Bu administration at three distinct dosages in inducing ovarian degeneration. Two-way ANOVA for follicle counts in the optimal dose group (CTX 100 mg/kg + Bu 20 mg/kg) at day 21 and during 3- and 4-week recovery showed significant effects of follicle type and group (*p* < 0.05), but no significant interaction. Both recovery groups exhibited persistently reduced primordial, primary, and antral follicles, with sustained atresia, confirming the stability of the POF model up to 4 weeks post-induction (Figure 5). These statistical outcomes are concordant with the histomorphological evidence observed in ovarian sections, further supporting the validity of the model.

**Figure 6:**
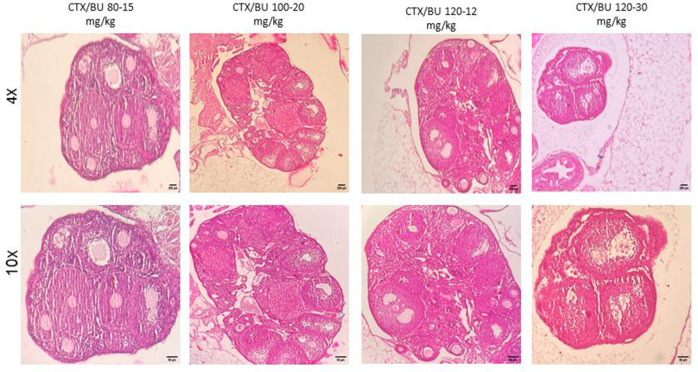
**POF in NMRI female 6-8 weeks mice.** Dose-response of ovarian follicles destruction by Cyclophosphamide (CTX: 80, 100, 120mg/Kg) and Busulfan (Bu: 15,20,12,30mg/Kg) for 3 weeks, observed at different magnifications (4× = 40×, 10× = 100×).

**Figure 7:**
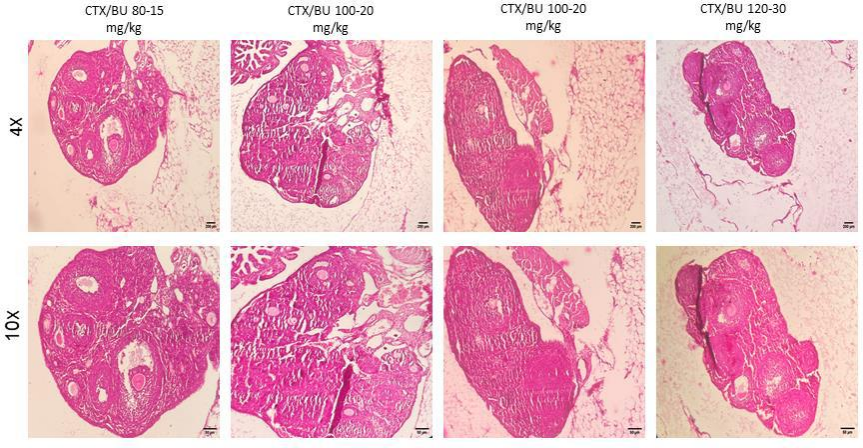
**POF failure (POF) in NMRI female 6-8 weeks mice.** Dose-response of ovarian follicles destruction by Cyclophosphamide (CTX: 80, 100, 120mg/Kg) and Busulfan (Bu: 15, 20, 12, 30 mg/Kg) for 4 weeks, observed at different magnifications (4× = 40×, 10× = 100×).

**Figure 8:**
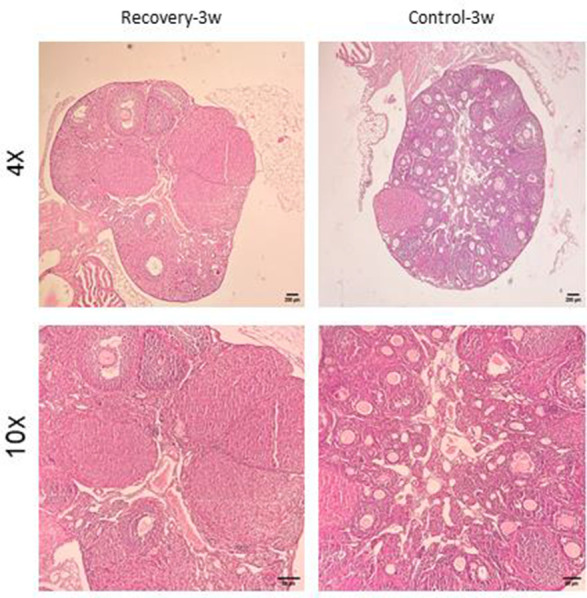
**Confirmation of natural recovery after 3 weeks of establishment POF model in NMRI female 6-8weeks mice.** Histological confirmation of ovarian POF phenotype three weeks after induction with CTX (100 mg/kg) + Bu (20 mg/kg), showing reduced primordial follicle reserve, increased atretic follicles, and widespread granulosa cell apoptosis.

**Figure 9:**
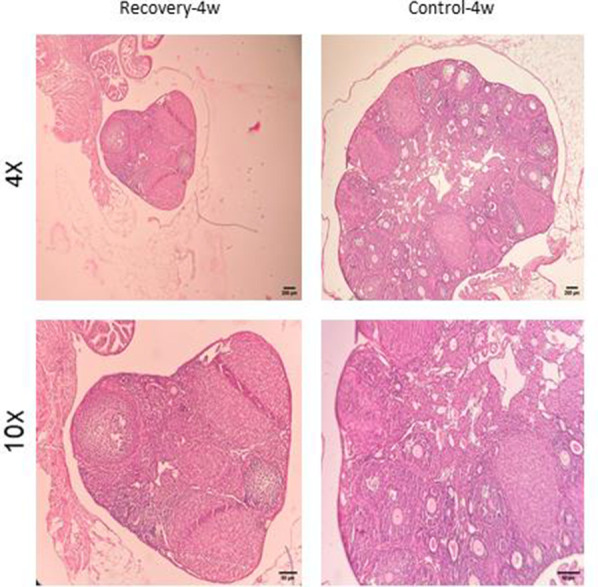
**Confirmation of natural recovery after 4 weeks of establishment POF model in NMRI female 6-8weeks mice.** Histological confirmation of POF phenotype at four weeks post-induction with CTX (100 mg/kg) + Bu (20 mg/kg). Findings demonstrate persistent follicular depletion and stromal atrophy with no signs of natural follicular recovery.

Histological features (Figures 6–9) characteristic of the induction of POF were evident, including oocyte degeneration, atrophy, and the absence of the zona pellucida in existing follicles, granulosa cell degeneration within follicles, disorganized granulosa cell layers, the formation of intercellular spaces, reduced ovarian volume and weight, and apoptotic indicators such as pyknotic nuclei in granulosa cells. These histopathological markers substantiate the establishment of a POF model, which was also discernible in the ovarian tissues of mice treated with group 2 dosages, although with lesser degrees of tissue degeneration, thereby offering more favorable conditions for biochemical and molecular analyses of ovarian tissue. Consequently, based on comprehensive histological assessments and natural recovery evaluations, a dosage of (CTX 100 mg/kg + Bu 20 mg/kg) is considered optimal for inducing a POF model that is conducive to biochemical and molecular studies, as well as for investigating the effects of various treatments on ovarian function in POF NMRI mice. Histological examination of the ovarian sections, stained with Hematoxylin-Eosin (HE), revealed notable alterations in follicle morphology and structure. The treated groups exhibited an increased incidence of atretic and apoptotic granulosa cells, indicating oocyte and follicular atresia and cellular apoptosis. Among the different dosage groups, the combination of (CTX 100 mg/kg + Bu 20 mg/kg) was identified as the optimal dose for inducing POF. This dose (CTX 100 mg/kg + Bu 20 mg/kg) treated mice showed a gradual process of follicle decline; these conditions were similar to POF in women. Moreover, this dosage paradigm maintained the structural and functional integrity of the ovarian stroma, thereby preserving tissue viability for the subsequent mechanistic analyses. Higher doses resulted in more histological atretic changes and prolonged impairment of ovarian function, whereas lower dose (80 mg/kg bw CTX and 15 mg/kg bw Bu) were less effective in establishing the POF model (Figures 6, 7). In addition, histological observations confirmed the stability of the optimal dose regimen and the maintenance of the POF phenotype throughout the entire observation period. Despite the absence of any additional interventions, no folliculogenesis or hormonal recovery was observed. Specifically, primordial, primary, and antral follicles remained significantly depleted (Figures 8, 9). Moreover, hormonal analysis demonstrated persistent reductions in AMH and E2 accompanied by elevated FSH levels (p < 0.01 vs. control), further confirming the stability of the model and the absence of ovarian regeneration.

### Effects of CTX/BU (100-20) on AMH, FSH and E2 levels

In the assessment of natural recovery and POF model stability in optimal dose, mice were left untreated for 3 and 4 weeks after POF induction. Suppressed serum of E2 and AMH and elevated FSH concentrations were considered as the parameters that convey the POF model stability 3 weeks after induction (Table 1).

**Table 1:** Hormonal analysis (control vs. optimal POF model).

**Hormone**	**Control (Mean ± SD)**	**POF (100/20) (Mean ± SD)**	**p-value**
AMH (ng/mL)	3.57 ± 0.7	1.18 ± 0.5	< 0.05
E2 (pg/mL)	76.15 ± 8.5	14.89 ± 2.9	< 0.01
FSH (mIU/mL)	1.99 ± 0.5	5.49 ± 0.6	< 0.01

**Figure 10:**
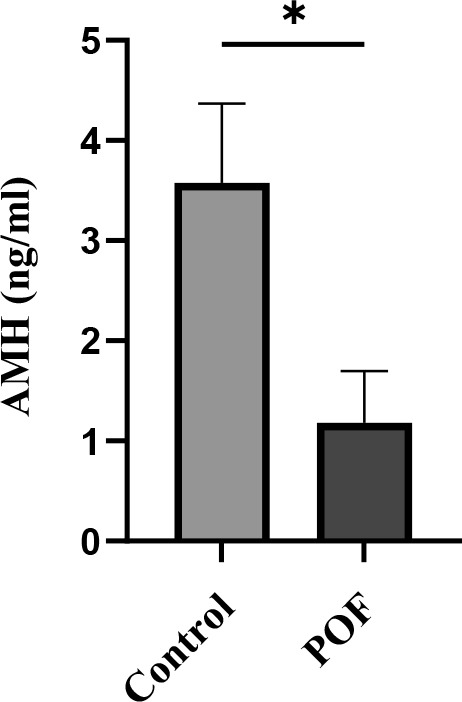
**Serum AMH levels measured 3 weeks after induction of POF in CTX (100 mg/kg)/Bu (20 mg/kg) - treated mice compared with control group.** Data are presented as mean ± SD; p < 0.01 versus control.

Serum AMH measurement revealed that in the control group the hormone level was 3.57 ± 0.7 ng/mL, whereas in the group treated with the optimal dose of CTX (100 mg/kg) + Bu (20 mg/kg) it was significantly reduced (1.18 ± 0.5 ng/mL; p < 0.05). This significant difference indicates persistent depletion of the follicular reserve and stability of the POF model up to three weeks after induction (Figure 10).

**Figure 11:**
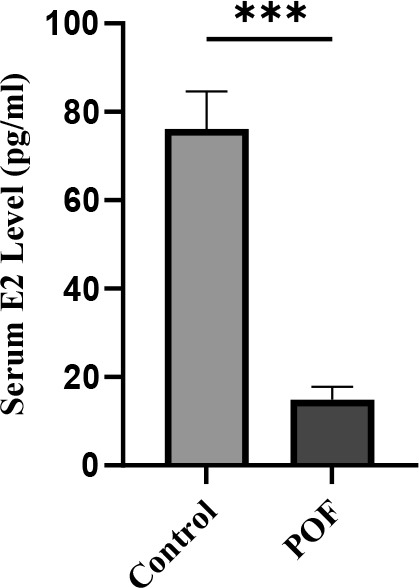
**Serum estradiol (E2) levels measured 3 weeks after POF induction in CTX (100 mg/kg)/Bu (20 mg/kg)-treated mice compared with control group.** Data are presented as mean ± SD; p < 0.01 versus control.

Serum E2 levels were markedly reduced in the CTX (100 mg/kg) + Bu (20 mg/kg)-treated group compared with controls (14.89 ± 2.9 vs. 76.15 ± 8.5 pg/mL; p < 0.01). This significant decline in E2 further confirms the induction of ovarian insufficiency and disruption of endocrine function in the POF model (Figure 11).

**Figure 12:**
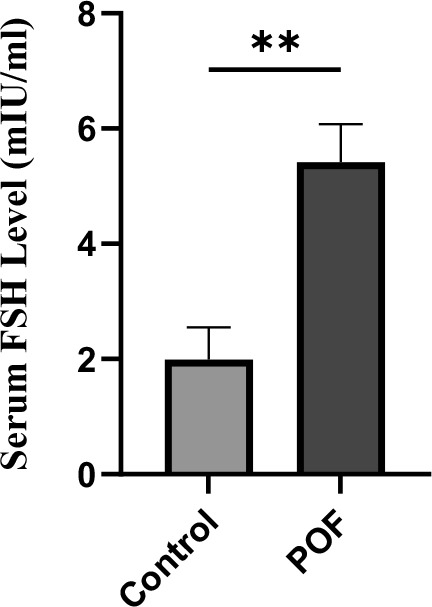
**Serum FSH levels measured 3 weeks after induction of POF in CTX (100 mg/kg)/Bu (20 mg/kg)-treated mice compared with control group.** Data are presented as mean ± SD; p < 0.01 versus control.

Serum FSH levels were significantly elevated in the CTX (100 mg/kg) + Bu (20 mg/kg)-treated group compared with controls (5.42 ± 0.6 vs. 1.99 ± 0.5 mIU/mL; p < 0.01). This endocrine alteration, in combination with decreased AMH and E2, is consistent with the hormonal signature of POF, confirming the stability of the induced POF model (Figure 12).

This hypergonadotropic hypoestrogenism profile—consistent with the endocrine signature of POF—corroborates the histopathological evidence of follicular depletion maintenance and POF stability until at least six weeks after IP injection of CTX/Bu.

## DISCUSSION

POF is a severe disorder affecting female fertility and can be caused by various factors [23]. One common way to induce POF is through chemotherapeutic agents such as CTX and Bu. which reduce primordial follicles and increase atretic follicles in the ovary, leading to POF [24]. Given the variability in concentration and duration of treatment across studies, we investigated the effects of CTX and Bu on inducing a POF model in mice. Our research aimed to establish an effective POF model using these chemotherapeutic agents, ensuring no recovery in ovarian function for 3–4 weeks, which would allow for various treatments. The selection of a three-week interval for recovery assessment, hormonal profiling, and body-weight monitoring in this study is biologically justified, as the natural course of folliculogenesis in mice—from the primordial to the antral stage—takes approximately 21 days. Accordingly, the use of a three-week endpoint provides a rational timeframe to evaluate the stability of the POF model [32].

Our results demonstrate that we established an optimized and cost-effective model in the mentioned strain of mice, accompanied by a gradual reduction in follicular reserve and hormonal changes (decreased AMH and E2 with increased FSH) resembled the typical endocrine profile observed in women with POF. According to recent clinical guidelines, the diagnosis of POF is primarily recommended to be based on persistently elevated FSH levels. In our study, a reduction in AMH was detected three weeks after model induction, which reflects the importance of considering the folliculogenesis interval in mice (~21 days) and further demonstrates the stability of the model. Collectively, these data indicate that our optimized protocol with CTX 100 mg/kg + Bu 20 mg/kg establishes a stable model of POF, which is highly suitable for investigating fertility-preserving interventions and exploring endocrine–follicular interactions during ovarian injury and recovery [33, 34].

Additionally, maintaining the model for 3 to 4 weeks after induction could provide a suitable option for biochemical analyses and other pharmacological interventions. In this regard, other studies have also been conducted.

Studies have indicated that the CHEK2 pathway is a key regulator of primordial oocyte elimination after chemotherapy and radiotherapy, and selective inhibition of this pathway can result in follicle preservation. These findings are consistent with our histological studies, which show extensive apoptosis of oocytes, granulosa cells, and atresia [35].

Kawano M et al. showed that granulosa cell death is the major factor in the reduction of follicular reserve and subsequent fertility reduction after exposure of mice to a combination of CTX (100 mg/kg body) and Bu (8.75 mg/kg) [36].

A recent study by Tang et al. conducted on rat models is consistent with our findings, demonstrating that a combination of CTX and Bu is a highly effective method for inducing POF. However, a fundamental discrepancy in the optimal dose for induction exists between our research and theirs. This difference may be attributed to a number of factors, most notably the divergent objectives and implementation protocols of the two studies. Specifically, the study by Tang et al. focused on a comparative evaluation of four distinct drug combinations. In contrast, our research was designed to optimize the dose of a single, highly effective.

Crucially, their findings did not account for the phenomenon of natural recovery following induction, a variable that was a central component of our dose selection criteria. Furthermore, variations in the specific strain of the rat species utilized in each study could also contribute to the observed differences in dose-response, underscoring the critical importance of species-specific protocols in POF research [27].

In another study conducted with the aim of measuring and comparatively evaluating several models of POF induction in rodents, body weight, related serum hormones, and ovarian histopathology were examined. In the group treated with CTX at a dose of 120, weight gain was slowed, and ovarian damage was observed, which aligned with our results in the combined groups. However, the Bu group did not show clear evidence of the POF phenotype, indicating that each compound has its own unique characteristics and mechanisms. Depending on the strain and duration of exposure to the compound, as well as the research objective, which could be a mechanism comparison or preventive study, the selection of the compound and dose plays an important role. What was studied and examined in our research was the long-term impact of the specified doses on ovarian tissue and the survival of the mice [37].

In a study, old female CD-1 mice received CTX and Bu intraperitoneally (120-12). The results of this study successfully confirmed and induced a mouse model related to POF failure, which was in line with our study and one of the selected doses for our project. However, considering the strain of mice in our study, the response was different from the present study. This underscores the influence of strain and breed on the outcomes derived from the induction of this model. The mention in the aforementioned article that treated mice exhibited reduced motor activity approximately 3 days after injection could support this observation, whereas our study found that NMRI mice showed no changes in activity or motor weakness [23].

In a study aimed at examining the effect of berberine on the ovaries of female mice induced with CTX and Bu in a model of POF, Bu was diluted in DMSO in the model used in this study, which is commonly employed in other studies for model induction. DMSO has systemic inflammatory/toxic effects and can therefore interact with the drug/intervention, affecting the results. For this reason, according to IACUC guidelines, it is recommended that in cases where this compound is used as a solvent, the DMSO control should be considered separately from the healthy control to enhance the accuracy of the results. For this reason, our careful selection of the company for drug preparation and the use of normal saline as a solvent for both compounds aim to increase the reliability of the obtained results [26].

In a separate study using combined CTX and Bu, investigators showed that administering CTX/BU for ≥2 weeks produced a persistent ovarian-insufficiency phenotype that did not spontaneously recover within two weeks after treatment cessation [10], a conclusion echoed by a recent methodological review [38]. These observations broadly align with our results; however, the severity of chemotherapy-based models is modulated by the number of injections, animal age, and genetic background (strain), and may also be influenced by vehicle choice. Consistent with this, when inducing POF in NMRI mice we systematically titrated CTX and Bu and observed dose-dependent ovarian effects. While higher doses consistently generated a POF phenotype, they exhausted the entire ovarian reserve and induced widespread granulosa-cell apoptosis followed by oocyte loss—an extreme less representative of many clinical POF cases, in which a limited follicle pool typically persists. By contrast, CTX 100 mg/kg plus Bu 20 mg/kg yielded the most informative histopathology—preserving a small primordial-follicle reserve while reproducing characteristic atresia—thereby most closely approximating clinical POF for the purposes of our study. A key limitation of this work was budgetary constraints, which restricted comprehensive endocrine monitoring. Consequently, following the identification of the optimal dose, hormonal analyses were performed only in this group for validation purposes, while assessments across the other experimental cohorts could not be carried out due to financial limitations.

In addition, direct monitoring of the estrous cycle through vaginal cytology was not performed. Such data could have complemented the histological and hormonal findings by providing dynamic insights into ovarian function and cyclicity.

For future directions, this model may serve as a platform for evaluating reproductive functions, including mating behavior analysis and pregnancy outcome tracking, as well as interventional studies aimed at improving ovarian function following optimal-dose induction.

## Conclusion

The development of reliable animal models for POF is critical for elucidating pathogenic mechanisms and evaluating potential therapeutic interventions. Our findings demonstrate that a single intraperitoneal coadministration of CTX (100 mg/kg) and Bu (20 mg/kg) induces a stable POF phenotype in NMRI mice within three weeks post-treatment, characterized by:

- Persistent ovarian dysfunction (no natural recovery observed during the 3-week post-induction period),
- Reproducible endocrine disruption (elevated FSH, suppressed AMH/E2), and
- Controlled toxicity (no significant increase in mortality versus controls).

This optimized protocol provides a cost-effective and technically accessible model system for:

- Investigating molecular pathways underlying chemotherapy-induced ovarian failure
- Screening novel therapeutic agents for fertility preservation
- Studying accelerated ovarian aging processes

The model’s phenotypic stability (≥3 weeks), combined with its clinical mimicry of human POF, thus making it a valuable preclinical tool for reproductive research.
